# Noxa upregulation by oncogenic activation of MEK/ERK through CREB promotes autophagy in human melanoma cells

**DOI:** 10.18632/oncotarget.2616

**Published:** 2014-10-21

**Authors:** Yi Lun Liu, Fritz Lai, James S. Wilmott, Xu Guang Yan, Xiao Ying Liu, Qi Luan, Su Tang Guo, Chen Chen Jiang, Hsin-Yi Tseng, Richard A. Scolyer, Lei Jin, Xu Dong Zhang

**Affiliations:** ^1^ School of Biomedical Sciences and Pharmacy, The University of Newcastle, NSW, Australia; ^2^ Discipline of Pathology, The University of Sydney, and Tissue Pathology and Diagnostic Oncology, Royal Prince Alfred Hospital, Sydney, NSW, Australia; ^3^ Department of Molecular Biology, Shanxi Cancer Hospital and Institute, Taiyuan, Shanxi, China; ^4^ School of Medicine and Public Health, The University of Newcastle, NSW, Australia

**Keywords:** Noxa, Autophagy, MEK/ERK, CREB, Melanoma

## Abstract

Reduction in the expression of the anti-survival BH3-only proteins PUMA and Bim is associated with the pathogenesis of melanoma. However, we have found that the expression of the other BH3-only protein Noxa is commonly upregulated in melanoma cells, and that this is driven by oncogenic activation of MEK/ERK. Immunohistochemistry studies showed that Noxa was expressed at higher levels in melanomas than nevi. Moreover, the expression of Noxa was increased in metastatic compared to primary melanomas, and in thick primaries compared to thin primaries. Inhibition of oncogenic BRAF^V600E^ or MEK downregulated Noxa, whereas activation of MEK/ERK caused its upregulation. In addition, introduction of BRAF^V600E^ increased Noxa expression in melanocytes. Upregulation of Noxa was due to a transcriptional increase mediated by cAMP responsive element binding protein, activation of which was also increased by MEK/ERK signaling in melanoma cells. Significantly, Noxa appeared necessary for constitutive activation of autophagy, albeit at low levels, by MEK/ERK in melanoma cells. Furthermore, it was required for autophagy activation that delayed apoptosis in melanoma cells undergoing nutrient deprivation. These results reveal that oncogenic activation of MEK/ERK drives Noxa expression to promote autophagy, and suggest that Noxa has an indirect anti-apoptosis role in melanoma cells under nutrient starvation conditions.

## INTRODUCTION

A characteristic of human melanoma is constitutive activation of the MEK/ERK signaling pathway [1, 2]. Identification of oncogenic mutations of BRAF (with the most common mutation being a glutamic acid for valine substitution at position 600 (BRAF^V600E^)) as the primary driver of aberrant activation of the pathway in melanoma has led to development of mutant BRAF inhibitors in the treatment of the disease [1, 3-5]. Although these inhibitors have achieved unprecedented response rates in melanoma patients, the benefits are often of limited duration [1, 3-5]. This is closely related to resistance of melanoma cells to apoptosis, as induction of apoptosis is a major determinant of long-term responses of BRAF^V600E^ melanoma cells to mutant BRAF inhibitors [3-5]. Similarly, responses of melanoma cells to inhibitors of MEK, the downstream protein kinase that activates ERK, are also closely associated with their sensitivity to induction of apoptosis [6, 7].

Bcl-2 family proteins play a central role in regulation of apoptosis [8, 9]. According to their biochemical structures and biological functions, they are largely divided into pro-survival proteins including Bcl-2, Mcl-1, Bcl-X_L_, and A1, BH3-only anti-survival proteins such as Bid, Bad, Bim, PUMA, and Noxa, and their effectors, the multidomain anti-survival proteins Bax and Bak [8, 9]. Activation of BH3-only proteins are essential in induction of apoptosis as they act as intracellular “death ligands” to activate Bax and Bak leading to mitochondrion damage by displacing them from pro-survival Bcl-2 family members [8, 9]. Deregulated expression of Bcl-2 family proteins either as consequences of genetic alterations or resulting from environmental stimulations contributes to the pathogenesis of various types of cancers including melanoma [10, 11]. For example, the expression of Mcl-1 increases with melanoma progression and is associated with poor patient prognosis [10]. In contrast, the BH3-only proteins PUMA and Bim are frequently reduced in melanomas, which is also associated with poor survival of patients [12, 13].

Besides regulation of apoptosis, Bcl-2 family proteins play important roles in regulating autophagy, an evolutionarily conserved process through which organelles and proteins are sequestered into autophagic vesicles (autophagosomes) within the cytosol [14-17]. Autophagosomes fuse with lysosomes to form autolysosomes leading to degradation of intracellular contents [15-17]. Autophagy is virtually a pro-survival mechanism that recycles cellular constituents to maintain nutrient supply in cells under stresses such as nutrition deprivation [15, 17]. However, excessive or prolonged autophagy leads to cell death [15, 17]. In the pathogenesis of cancer, autophagy is regarded as a double-edged sword as it functions to promote or inhibit cancer development and progression in a stage- and context-dependent manner [18, 19]. Nevertheless, increasing evidence has shown that autophagy in general promotes cancer cell survival under various cellular stresses [18-21].

Pro-survival Bcl-2 family proteins negatively regulate autophagy by binding to beclin-1, which plays an important role in autophagosome formation through forming complexes with Phosphatidylinositol 3-kinase catalytic subunit type 3 (PIK3C3)/mammalian vacuolar protein sorting 34 homologue (hVps34) that is a nutrient-regulated lipid kinase [14, 16, 22]. Binding of Bcl-2, Bcl-X_L_, or Mcl-1 with beclin-1 interferes with the association of beclin-1 with PIK3C3/hVps34 thus inhibiting its autophagy-inducing potential [14, 16, 23]. Beclin-1 is a BH3-only protein and its association with pro-survival Bcl-2 family proteins is competitively inhibited by other BH3-only proteins of the Bcl-2 family [14, 16]. Therefore, anti-survival BH3-only Bcl-2 family proteins can activate autophagy through freeing beclin-1. In this regard, it is of note that binding of BH3-only proteins to pro-survival Bcl-2 proteins is highly selective [24]. For example, while Bim can bind to all pro-survival proteins of the family, Noxa can only bind to Mcl-1 and A1 [24].

In this study, we have found that the BH3-only protein Noxa is commonly upregulated in melanomas, and that this is associated with melanoma development and progression. We report here that the increase in Noxa expression is driven by oncogenic activation of MEK/ERK signaling through the transcription factor cAMP responsive element binding protein (CREB), and that Noxa contributes to constitutive activation of autophagy in melanoma cells. In addition, we demonstrate that Noxa is necessary for induction of autophagy that delays apoptosis in melanoma cells under nutrient starvation conditions.

## RESULTS

### Noxa is commonly upregulated in human melanoma

**Figure 1:**
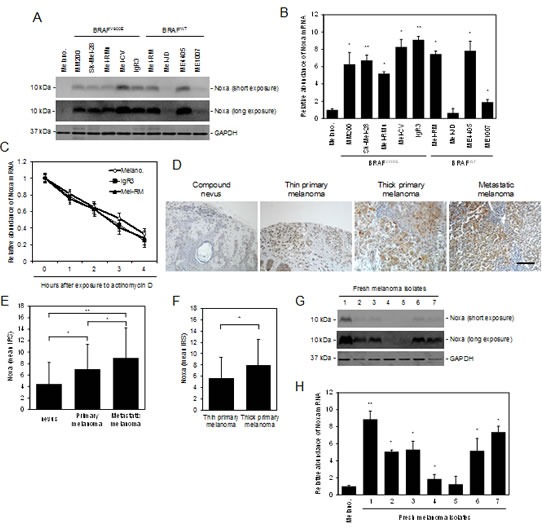
Noxa is upregulated in human melanoma (A) Whole cell lysates from melanocytes and melanoma cells were subjected to western blot analysis. Data shown are representative of three individual experiments. (B) Total RNA from melanocytes and melanoma cells were subjected to qPCR analysis. The relative abundance of Noxa mRNA expression in melanocytes was arbitrarily designated as 1 (n=3, mean ± S.E.M.). *P < 0.05, ** *P* < 0.01, Student's *t*-test. (C) Total RNA from melanocytes and melanoma cells treated with actinomycin D (100 ng/ml) for the indicated periods was subjected to qPCR analysis. The relative abundance of Noxa mRNA expression of each cell line before treatment was arbitrarily designated as 1 (*n*=3, mean ± S.E.M.). (D) Representative microphotographs of IHC staining of Noxa in melanocytic tumor sections. Scale bar, 100 μm. (E) (F) Quantitation of Noxa expression levels in melanocytic tumors. Data shown are mean immunoreactive score (IRS) ± S.E.M. of three individual experiments. *P < 0.05, ** *P* < 0.01, Student's *t*-test. (G) Whole cell lysates from melanocytes and fresh melanoma isolates were subjected to western blot analysis. Data shown are representative of three individual experiments. (H) Total RNA from melanocytes and fresh melanoma isolates were subjected to qPCR analysis. The relative abundance of Noxa mRNA expression in melanocytes was arbitrarily designated as 1 (*n*=3, mean ± S.E.M.). *P < 0.05, ** *P* < 0.01, Student's *t*-test.

Melanoma development and progression is closely associated with downregulation of some anti-survival Bcl-2 family proteins, such as the BH3-only proteins PUMA and Bim [12, 13]. However, the other BH3-only protein Noxa appeared to be commonly upregulated in cultured melanoma cells compared to melanocytes (Figure 1A). While Noxa was not measurable in pooled melanocytes of three different lines (HEMa-LP, HEMn-DP, and HEMn-MP) by immunoblotting (pooled melanocytes were used to simplify analysis, as these melanocyte lines similarly did not express Noxa; Supplementary Figure S1), it was detected in all melanoma cell lines at various levels (Figure 1A). Similar to its protein expression, Noxa was commonly increased at the mRNA level in melanoma cell lines (Figure 1B). Nevertheless, the turnover rate of the Noxa mRNA in melanoma cells was similar to that in melanocytes as shown by actinomycin D-chase assays (Figure 1C), indicating that upregulation of the Noxa transcript in melanoma cells is due to a transcriptional increase rather than changes in its stability [25, 26]. Of note, although p53 is commonly involved in transcriptional upregulation of Noxa [27], ME4405 cells that were deficient in p53 and Sk-Mel-28 cells that harboured mutant p53 expressed Noxa at levels comparable to those carrying wild-type p53 (Figures 1A and B) [28, 29]. This suggests that p53 may not play a major role in upregulation of Noxa in melanoma cells under steady-state conditions.

We also examined the expression of Noxa in relation to melanoma development and progression by immunohistochemistry in TMAs constructed from 100 formalin-fixed paraffin-embedded (FFPE) melanocytic tumors (Supplementary Table 1) [30]. The results revealed that Noxa was detected in most nevi and melanomas (Figures 1D and E). However, its expression was upregulated in melanomas compared to nevi (Figures 1D and E). In addition, the levels of the expression were increased in metastatic melanomas compared to primary melanomas (Figures 1D and E), and in thick primaries compared to thin primaries (Figures 1D and F). These results suggest that Noxa is upregulated along with melanoma development and progression. In support, Noxa was detected in a panel of fresh metastatic melanoma isolates at the protein and mRNA levels (Figures 1G and H).

### Activation of MEK/ERK drives Noxa expression in melanoma cells

**Figure 2:**
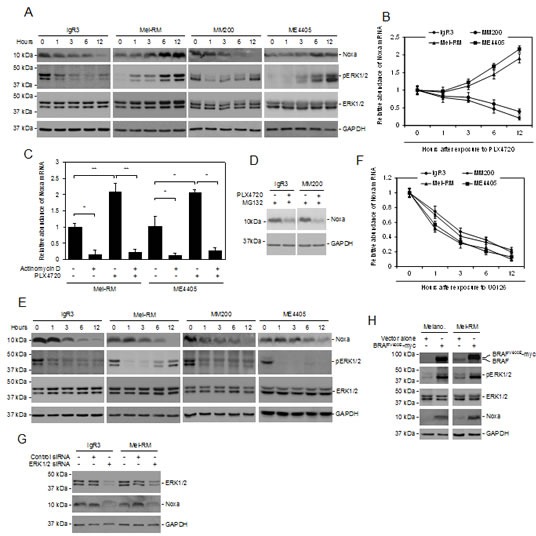
Noxa expression is driven by activation of MEK/ERK signaling in melanoma cells (A) Whole cell lysates from BRAF^V600E^ melanoma cells (IgR3 and MM200) and BRAF^WT^ melanoma cells (Mel-RM and ME4405) treated with the mutant BRAF-specific inhibitor PLX4720 (3μM) for indicated periods were subjected to western blot analysis. Data shown are representative of three individual experiments. (B) Total RNA from IgR3, MM200, Mel-RM, and ME4405 cells treated with PLX4720 (3μM) for the indicated periods were subjected to qPCR analysis. The relative abundance of Noxa mRNA expression of each cell line before treatment was arbitrarily designated as 1 (*n*=3, mean ± S.E.M.). (C) Mel-RM and ME4405 (BRAF^WT^) cells with or without pretreatment with actinomycin D (100 ng/ml) for 1 hour were treated with PLX4720 (3μM) for a further12 hours. Total RNA was subjected to qPCR analysis. The relative abundance of Noxa mRNA expression of each cell line without treatment was arbitrarily designated as 1 (*n*=3, mean ± S.E.M.). *P < 0.05, ** *P* < 0.01. Student's *t*-test. (D) IgR3 and MM200 (BRAF^V600E^) cells with or without pretreatment with the proteasome inhibitor MG132 (10μM) for 1 hour were treated with PLX4720 (3μM) for a further 6 hours. Whole cell lysates were subjected to western blot analysis. Data shown are representative of three individual experiments. (E) Whole cell lysates from IgR3, MM200, Mel-RM, and ME4405 cells treated with U0126 (20μM) for the indicated periods were subjected to western blot analysis. Data shown are representative of three individual experiments. (F) Total RNA from IgR3, MM200, Mel-RM, and ME4405 cells treated with U0126 (20μM) for the indicated periods was subjected to qPCR analysis. The relative abundance of Noxa mRNA expression of each cell line before treatment was arbitrarily designated as 1 (*n*=3, mean ± S.E.M.). (G) Whole cell lysates from IgR3 (BRAF^V600E^) and Mel-RM (BRAF^WT^) cells transfected with the control or ERK1 plus MEK2 siRNA were subjected to western blot analysis. Data shown are representative of three individual experiments. (H) Whole cell lysates from melanocytes and Mel-RM (BRAF^WT^) cells transduced with BRAF^V600E^-myc constructs were subjected to western blot analysis. Data shown are representative of three individual experiments.

Since Noxa is downregulated by BRAF inhibitors in mutant BRAF melanoma cells [31], we tested whether activation of MEK/ERK signaling is essential for constitutive expression of Noxa in the cells. In keeping with previous reports [31]. Noxa was rapidly reduced at both the protein and mRNA levels in BRAF^V600E^ melanoma cells (IgR3 and MM200 cells) after treatment with the mutant BRAF inhibitor PLX4720 (Figures 2A and B). However, it was increased by PLX4720 in Mel-RM and ME4405 cells that harboured wild-type BRAF (BRAF^WT^) (Figures 2A and B). These contrasting effects on the expression of Noxa were associated with differential impacts of PLX4720 on activation of ERK1/2, which was, as anticipated, inhibited in BRAF^V600E^, but enhanced in BRAF^WT^, melanoma cells (Figures 2A and B) [32-34]. Regulation of Noxa expression by PLX4720 appeared to be mediated by a transcriptional mechanism, in that the addition of actinomycin D abolished the increase in the Noxa transcript in Mel-RM and ME4405 cells treated with the inhibitor (Figure 2C). In support, the Noxa protein levels in BRAF^V600E^ melanoma cells treated with PLX4720 in the presence of the proteasome inhibitor MG132 remained significantly lower compared to those without exposure to the BRAF inhibitor (Figure 2D) [35].

To confirm that downregulation of Noxa by PLX4720 in BRAF^V600E^ melanoma cells is due to inhibition of the MEK/ERK pathway, we treated IgR3 and MM200 (BRAF^V600E^), and Mel-RM and ME4405 (BRAF^WT^) cells with the MEK inhibitor U0126. Indeed, inhibition of MEK downregulated the Noxa protein and mRNA in both BRAF^V600E^ and BRAF^WT^ melanoma cells (Figures 2E and F). This was associated with reduction in ERK activation irrespective of the BRAF mutational status of the cells (Figures 2E and F). In line with this, knockdown of ERK1/2 with siRNA similarly caused downregulation of Noxa in both BRAF^V600E^ and BRAF^WT^ melanoma cells (Figure 2G).

To further consolidate that activation of the MEK/ERK pathway upregulates Noxa in melanocytic cells, we infected HEMn-MP human melanocytes with lentiviral constructs expressing BRAF^V600E^ (Figure 2H). Enforced expression of BRAF^V600E^ resulted in activation of ERK, which was associated with induction of Noxa (Figure 2H). Similarly, introduction of exogenous BRAF^V600E^ into BRAF^WT^ melanoma cells (Mel-RM) also resulted in upregulation of Noxa in association with activation of ERK (Figure 2H). Together, these results reiterate the importance of activation of MEK/ERK in driving Noxa expression in melanoma cells.

### CREB mediates transcriptional upregulation of Noxa by MEK/ERK signaling

**Figure 3:**
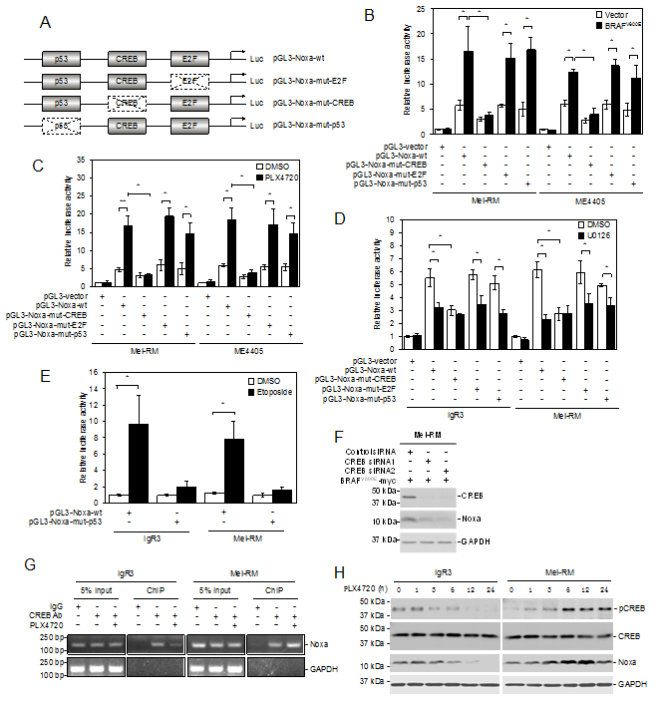
CREB mediates transcriptional upregulation of Noxa by MEK/ERK signaling in melanoma cells (A) A schematic illustration of the luciferase reporter constructs. Mutations in the p53, E2F, and CREB binding sites are indicated with dotted boxes.(B) Mel-RM and ME4405 (BRAF^WT^) cells were transiently transfected with the indicated pGL3-reporter constructs with or without co-transfection with a construct expressing BRAF^V600E^. Luciferase activity was measured 24 hours latter. The relative luciferase activity in cells transfected with pGL3-vector was arbitrarily designated as 1 (*n*=3, mean ± S.E.M.). *P < 0.05, Student's *t*-test. (C) Mel-RM and ME4405 (BRAF^WT^) cells were transiently transfected with the indicated pGL3-reporter constructs. Twenty-four hours later, cells were treated with PLX4720 (3μM) for a further 12 hours followed by measurement of luciferase activity. The relative luciferase activity in cells transfected with pGL3-vector was arbitrarily designated as 1 (*n*=3, mean ± S.E.M.). *P < 0.05, ** *P* < 0.01, Student's *t*-test. (D) IgR3 (BRAF^V600E^) and Mel-RM (BRAF^WT^) were transiently transfected with indicated pGL3-reporter constructs. Twenty-four hours later, cells were treated with U0126 (20μM) for a further 12 hours followed by measurement of luciferase activity. The relative luciferase activity in cells transfected with pGL3-vector was arbitrarily designated as 1 (*n*=3, mean ± S.E.M.). *P < 0.05, Student's *t*-test. (E) IgR3 and Mel-RM (wild-type p53) were transiently transfected with indicated pGL3-reporter constructs. Twenty-four hours later, cells were treated with etoposide (50μM) for a further12 hours followed by measurement of luciferase activity. The relative luciferase activity in cells transfected with pGL3-vector was arbitrarily designated as 1 (n=3, mean ± S.E.M.). *P < 0.05, Student's *t*-test. (F) Mel-RM (BRAF^WT^) cells stably transduced with BRAF^V600E^ were transfected with the control or CREB siRNA. Twenty-four hours later, whole-cell lysates were subjected to Western blot analysis. Data shown are representative of three individual experiments. (G) Formaldehyde-cross-linked chromatin of IgR3 (BRAF^V600E^) and Mel-RM (BRAF^WT^) cells with or without treatment with PLX4720 (3μM) for 12 hours was subjected to immunoprecipitation with an antibody against CREB. The resultant precipitates were subjected to PCR amplification using primers directed to the *Noxa* promoter. Data shown are representative of three individual experiments. (H) Whole-cell lysates from IgR3 (BRAF^V600E^) and Mel-RM (BRAF^WT^) cells with or without treatment with PLX4720 (3μM) for the indicated periods were subjected to western blot analysis. Data shown are representative of three individual experiments.

A number of transcription factors, including p53, E2F1 and CREB, are involved in transcriptional activation of Noxa in a cell type- and context-dependent manner [27, 36-38]. To examine the mechanism responsible for transcriptional upregulation of Noxa by MEK/ERK signaling, we introduced luciferase reporter constructs of the *Noxa* promoter with deletions of the known binding site for p53, E2F1, or CREB into BRAF^WT^ Mel-RM and ME4405 cells (Figure 3A and Supplementary Figure S2). Deletion of the CREB binding site, but not deletion of the binding site for p53 or E2F1, markedly inhibited the transcriptional activity of the *Noxa* promoter in Mel-RM and ME4405 cells with or without co-introduction of BRAF^V600E^ (Figure 3B). Similarly, deletion of the CREB binding site, but not deletion of the binding site for p53 or E2F1, inhibited the increase in the transcriptional activity in response to PLX4720 (Figure 3C). Moreover, deletion of CREB binding site did not cause any further reduction in the transcriptional activity of the Noxa promoter in BRAF^V600E^ IgR3 and BRAF^WT^ Mel-RM cells treated with U0126 (Figure 3D). Of note, deletion of the p53 binding site attenuated upregulation of Noxa by the DNA-damaging drug etoposide, consistent the role of p53 in DNA damage-mediated induction of Noxa (Figure 3E) [27]. Taken together, these results suggest that transcriptional upregulation of Noxa by MEK/ERK signaling is primarily mediated by CREB. In support, knockdown of CREB abolished upregulation of endogenous Noxa by ectopic expression of BRAF^V600E^ in Mel-RM cells (Figure 3F).

The role of CREB in regulation of Noxa by MEK/ERK signaling was further confirmed by its association with the *Noxa* promoter in melanoma cells regardless of their BRAF mutational status as shown by chromatin immunoprecipitation (ChIP) assays (Figure 3G). The association of CREB with the promoter of *Noxa* was however markedly reduced in BRAF^V600E^, but was increased in BRAF^WT^, melanoma cells by treatment with PLX4720 (Figure 3G). Noticeably, PLX4720 decreased CREB activation (phosphorylation) in BRAF^V600E^ melanoma cells (Figure 3H). In contrast, activation of CREB was increased by PLX4720 in BRAF^WT^ melanoma cells (Figure 3H).

### Activation of MEK/ERK drives autophagy in melanoma cells

**Figure 4:**
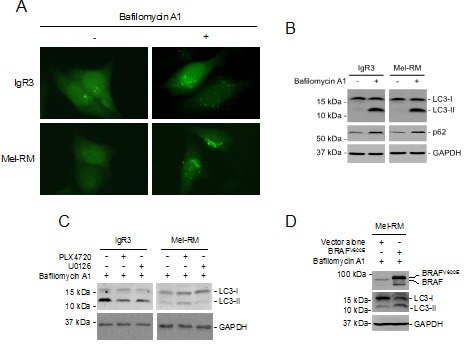
MER/ERK drives activation of autophagy in melanoma cells (A) GFP-LC3 constructs were transfected into IgR3 and Mel-RM cells. Twenty-four hours later, cells were treated bafilomycin A1 (100nM) for a further 2 hours. GFP-LC3 puncta formation was monitored using a fluorescence microscope. Data shown are representative of three individual experiments. (B) Whole-cell lysates from IgR3 and Mel-RM cells with or without treatment with bafilomycin A1 (100nM) for 2 hours were subjected to western blot analysis. Data shown are representative of three individual experiments. (C) Whole cell lysates from IgR3 (BRAF^V600E^) and Mel-RM (BRAF^WT^) cells with or without treatment with PLX4720 (3μM) or U0126 (20μM) for 24 hours in the presence of bafilomycin A1 (100nM) were subjected to western blot analysis. Data shown are representative of three individual experiments. (D) Mel-RM cells transfected with BRAF^V600E^ were treated with bafilomycin A1 (100nM) for 2 hours. Whole cell lysates were subjected to western blot analysis. Data shown are representative of three individual experiments.

Although hyperactivation oncogenic BRAF induces macroautophagy (hereafter referred to as autophagy) in melanoma cells [39], markers of autophagy activation, including conversion of LC3-I to LC3-II and aggregation (punctate staining) of GFP-LC3 that was stably introduced into cells by lentiviral transduction, could hardly be detected in BRAF^V600E^ as well as BRAF^WT^ melanoma cells (Figures 4A and B). However, inhibition of the autophagy flux at late stages by bafilomycin A1, which inhibits fusion of autophagosomes with lysosomes, resulted in accumulation of LC3-II and increases in p62, a receptor for cargos destined to be degraded by autophagy that would otherwise be decreased by lysosomal degradation of autophagosomes (Figure 4B) [40], in both BRAF^V600E^ and BRAF^WT^ melanoma cells. Thus, the autophagy flux is constitutively activated at low levels in melanoma cells irrespective of their BRAF mutational status.

To test whether constitutive activation of MEK/ERK signaling is essential for the autophagy activity in melanoma cells, we treated IgR3 and Mel-RM cells with U0126 before the addition of bafilomycin A1. U0126 abolished bafilomycin A1-triggered accumulation of LC3-II in both cell lines (Figure 4C). Similarly, PLX4720 inhibited the increase in LC3-II induced by bafilomycin A1 in IgR3 cells (Figure 4C). However, it further enhanced accumulation of LC3-II in Mel-RM cells (Figure 4C). These results suggest that the MEK/ERK signaling plays a critical role in driving basal autophagy activity in melanoma cells. In support, ectopic expression of BRAF^V600E^ in Mel-RM cells increased accumulation of LC3-II induced by bafilomycin A1 (Figure 4D).

### Noxa is necessary for MEK/ERK-driven autophagy in melanoma cells

**Figure 5:**
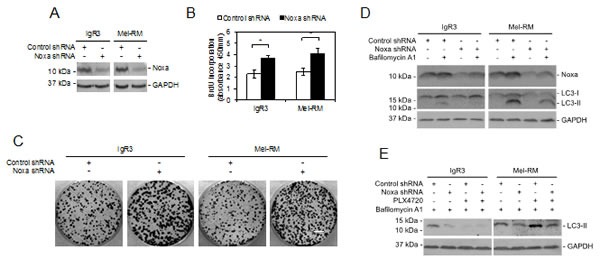
Noxa is necessary for MEK/ERK-driven autophagy in melanoma cells (A) Whole cell lysates from IgR3 and Mel-RM cells stably transduced with the control or Noxa shRNA were subjected to western blot analysis. Data shown are representative of three individual experiments. (B) IgR3 and Mel-RM cells stably transduced with the control shRNA or Noxa shRNA were subjected to cell proliferation assays using the BrdU incorporation method. Data shown are representative of three individual experiments. (*n*=3, mean ± S.E.M.). *P < 0.05, Student's *t*-test. (C) IgR3 and Mel-RM cells stably transduced with the control or Noxa shRNA were subjected to clonogenic assays. Data shown are representative of three individual experiments. Scale bar, 1cm. (D) IgR3 and Mel-RM cells stably transduced with the control or Noxa shRNA were treated with or without bafilomycin A1 (100nM) for 2 hours. Whole-cell lysates were subjected to western blot analysis. Data shown are representative of three individual experiments. (E) IgR3 and Mel-RM cells stably transduced with the control or Noxa shRNA were treated with PLX4720 (3μM) or U0126 (20μM) for 24 hours in the presence of bafilomycin A1 (100nM). Whole-cell lysates were subjected to western blot analysis. Data shown are representative of three individual experiments.

Noxa plays a role in induction of autophagy by oncogenic activation of RAS, which is an upstream kinase of RAF/MEK/ERK signaling [23]. Since Noxa is upregulated by activation of MEK/ERK, we examined whether Noxa is involved in induction of autophagy by MEK/ERK signaling. To this end, we stably knocked down Noxa in IgR3 and Mel-RM cells by shRNA (Figure 5A). Inhibition of Noxa resulted in increases in melanoma cell proliferation as shown in 5-bromo-2′-deoxyuridine (BrdU) incorporation and clonogenic assays (Figures 5B and C), suggesting that Noxa negatively regulates melanoma cell proliferation. On the other hand, it reduced the accumulation of LC3-II caused by bafilomycin A1, recapitulating the effect of U0126 on the autophagy flux in both IgR3 and Mel-RM cells, and the effect of PLX4720 in IgR3 cells (Figures 4C and 5D). Furthermore, knockdown of Noxa blocked accumulation of LC3-II in Mel-RM cells triggered by bafilomycin A1 in the presence of PLX4720 (Figure 5E). Collectively, these results indicate that Noxa is required by autophagy driven by MEK/ERK signaling in melanoma cells.

### Noxa is necessary for nutrient starvation-triggered autophagy in melanoma cells

**Figure 6:**
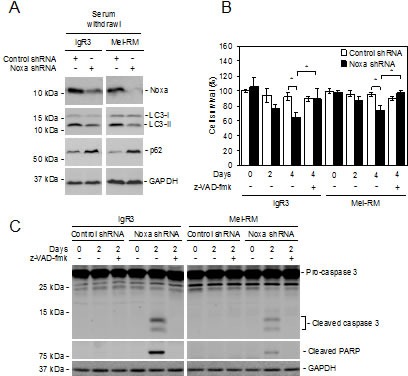
Noxa is necessary for nutrient starvation-triggered autophagy in melanoma cells (A) IgR3 and Mel-RM cells stably transduced with the control or Noxa shRNA were cultured in media with serum withdrawal for 2 days. Whole-cell lysates were subjected to western blot analysis. Data shown are representative of three individual experiments. (B) IgR3 and Mel-RM cells stably transduced with the control or Noxa shRNA were cultured in media with serum withdrawal for the indicated periods in the absence or presence of z-VAD-fmk (30μM). Cells were subjected to CellTiter-Glo cell viability assays. Data shown are representative of three individual experiments. (*n*=3, mean ± S.E.M.). *P < 0.05, Student's *t*-test. (C) IgR3 and Mel-RM cells stably transduced with the control or Noxa shRNA were cultured in media with serum withdrawal for 2 days in the absence or presence of z-VAD-fmk (30μM). Whole-cell lysates were subjected to western blot analysis. Data shown are representative of three individual experiments.

To further examine the role of Noxa in induction of autophagy in melanoma cells, we withdrew serum from the cultures of IgR3 and Mel-RM cells transduced with the control or Noxa shRNA. Knockdown of Noxa inhibited activation of autophagy in melanoma cells under serum starvation conditions as shown by reduced LC3-II and elevated p62 levels (Figure 6A). These results suggest that Noxa also plays a role in autophagy induced by nutrient starvation in melanoma cells. Of note, Noxa knockdown accelerated reduction in cell viability caused by serum starvation, which was largely due to induction of apoptosis as shown by caspase-3 activation and cleavage of its substrate PARP (Figures 6B and C). Furthermore, the general caspase inhibitor z-VAD-fmk inhibited cell death at relatively early stages after withdrawal of serum (Figures 6B and C) [41]. These results suggest that activation of autophagy mediated by Noxa delays apoptosis in melanoma cells induced by serum starvation.

## DISCUSSION

In this report, we present evidence that the BH3-only protein Noxa is upregulated by oncogenic activation of MEK/ERK signaling, and that Noxa plays an important role in regulation of autophagy in melanoma cells. While the increase in Noxa expression was associated with melanoma development and progression, inhibition of MEK/ERK signaling downregulated, and activation of the pathway upregulated, the expression of Noxa in melanoma cells. Moreover, introduction of oncogenic BRAF^V600E^ increased Noxa expression in melanocytes. Our results also showed that Noxa was a driver of constitutive activation of autophagy, and in particular, was necessary for induction of autophagy that delayed apoptosis in melanoma cells under nutrient starvation conditions.

BH3-only proteins of the Bcl-2 family play an important role in activation of mitochondrial apoptosis signaling [8, 9]. As such, downregulation of BH3-only proteins is a common mechanism by which cancer cells evade apoptosis [10, 11]. In particular, both PUMA and Bim are commonly reduced in human melanomas, which is associated with melanoma development and progression [12, 13]. Therefore, the finding that Noxa is frequently upregulated in melanomas, which is also associated with the pathogenesis of the disease is intriguing, as this suggests that Noxa may have a pro-survival role in melanoma cells while its apoptosis-inducing potential is kept in check. Since Mcl-1 that can bind to and inhibit Noxa is also increased with melanoma progression [10, 24], and the ability of Noxa to trigger apoptosis is determined by a balance between its expression levels and the levels of Mcl-1 [37], it seems that high expression of Mcl-1 is sufficient to enable melanoma cells to withstand similarly high levels of Noxa. In support of this, knockdown of Mcl-1 induces, albeit moderately, apoptosis in melanoma cells [42].

Is there any functional significance of high expression of Noxa in melanoma cells? Given that evasion of apoptosis is a well-established hallmark of cancer [43, 44], it is unlikely that cancer cells selectively upregulate a potentially lethal protein as a bystander. Indeed, our results showed that Noxa was involved in constitutive activation of autophagy in melanoma cells. Although the extent of autophagy in cultured melanoma cells was low, it is known that autophagy occurs commonly in cancer cells *in vivo* [45, 46]. Cells in a developing solid cancer are often under nutrient starvation conditions due to lack of sufficient blood supply [47, 48]. On the other hand, the fast dividing rate of cancer cells may also directly uncouple nutrient demand and supply [49]. It is conceivable that the increased Noxa expression in the face of high levels of Mcl-1 may drive finely-controlled autophagy that recycle damaged organelles and unwanted proteins as a supplementary nutrient source without induction of apoptosis. Indeed, we found that Noxa was required for induction of autophagy that delayed occurrence of apoptosis in melanoma cells under serum starvation conditions. Consistent with this, autophagy protects cells against apoptosis in many experimental systems [18, 19, 47]. Collectively, these results suggest that, instead of being simply a pro-apoptosis protein, Noxa functions as a safety guard that not only supplements nutrient supply but also has a pro-survival role through activation of autophagy in melanoma cells under starvation conditions. However, knockdown of Noxa enhanced proliferation of melanoma cells cultured under nutrient (serum)-sufficient conditions. This is conceivably caused by reduction in autophagy activity resulting from deficiency in Noxa that causes accumulation of p62, thus leading to increased melanoma cell proliferation, as autophagy deficiency has been recently reported to promote cell proliferation through p62 [50].

The finding that oncogenic activation of MEK/ERK signaling is responsible for Noxa upregulation in melanoma cells is similarly intriguing, as oncogenic activation of the MEK/ERK pathway is known to be a major driver of the pathogenesis of melanoma that suppresses apoptosis [1, 2, 6, 7]. Nevertheless, the pathway also plays a major role in upregulation of Mcl-1 in melanoma cells [6, 51]. It appears that a major role of Mcl-1 upregulation by oncogenic activation of MEK/ERK is to neutralize the apoptosis-inducing potential of Noxa to ensure cell survival thus making possible for its autophagy-inducing function to operate. Indeed, Noxa contributes to induction of apoptosis of melanoma cells when Mcl-1 is inhibited [42, 52]. Moreover, downregulation of Noxa has been recently reported to counteract apoptosis induction by BRAF inhibitors in mutant BRAF melanoma cells [31].

Similar to other pro-survival Bcl-2 family proteins such as Bcl-2 and Bcl-X_L_, Mcl-1 can bind to beclin-1 that is a BH3-only protein and thus inhibits beclin-1-dependent induction of autophagy [23]. Competitive binding of other BH3-only proteins of the Bcl-2 family to the pro-survival proteins frees beclin-1 leading to activation of autophagy [14-17]. Indeed, Noxa is responsible for oncogenic RAS-induced autophagy through displacing Mcl-1 from beclin-1 in other types of cells [23]. It is conceivable that Noxa may similarly activate autophagy triggered by oncogenic activation of MEK/ERK through displacement of Mcl-1 from beclin-1, and that a dynamic balance between the Mcl-1/Noxa and Mcl-1/beclin-1 complexes contributes to regulation of autophagy and apoptosis in melanoma cells, in particular, when cells are under stress conditions such as nutrient starvation.

As a protein with a fast turnover rate, Noxa is frequently regulated by posttranslational mechanisms [53, 54]. Nevertheless, upregulation of Noxa by MEK/ERK signaling in melanoma cells appeared largely due to a transcriptional increase, in that the Noxa mRNA was also increased similar to its protein, and that the turnover rate of the mRNA in melanoma cells remained similar to that in melanocytes. Although a number of transcription factors such as p53 and E2F1 are involved in transcriptional activation of Noxa [27, 36], we found that the transcription factor CREB is responsible for upregulation of Noxa by oncogenic activation of MEK/ERK in melanoma cells, whereas neither p53 nor E2F1 was involved. In support, CREB activation appeared to be increased by activation of MEK/ERK signaling. CREB is overexpressed and activated in melanomas with disease progression, which is often associated with activation of the PI3K/Akt signaling pathway [38, 55, 56]. Consistent with our finding that MEK/ERK signaling also enhances activation of CREB, a recent study has demonstrated by analysis of biopsies from BRAF^V600E^ melanoma patients that activation of CREB was supressed by inhibition of mutant BRAF or MEK but restored in relapsing melanomas [57]. CREB-mediated upregulation of Noxa is known to be critical to break the balance of Noxa/Mcl-1 in induction of apoptosis by some chemotherapeutic drugs [37]. p53-independent induction of Noxa by CREB has also been previously reported [38].

In summary, we have demonstrated that oncogenic activation of MEK/ERK signaling upregulates the expression of Noxa, which plays an important role in activation of autophagy, in particular, in melanoma cells under nutrient starvation conditions. These results not only reveal an unexpected role of Noxa in protection of melanoma cells from apoptosis through activation of autophagy in a context-dependent manner, but also uncover a novel mechanism by which oncogenic activation of MEK/ERK promotes the pathogenesis of melanoma. In addition, our results support the notion that inhibition of autophagy may improve the therapeutic efficacy of inhibitors of BRAF and MEK in the treatment of melanoma [58, 59].

## METHODS

### Cell lines and human tissues

Human melanoma cell lines were cultured in DMEM containing 5% FCS as described previously [60]. Human melanocyte lines (HEMa-LP, HEMn-DP and HEMn-MP) were purchased from Banksia Scientific (Bulimba, QLD, Australia) and cultured in melanocyte medium (Gibco, Invitrogen, Australia). Human fresh melanoma isolates were prepared from fresh surgical specimens according to the published method [61]. DNA for cell line authentication was extracted from all cell lines while in culture for this study. Individual cell line authentication was confirmed using the AmpFISTR Identifiler PCR Amplification Kit from Applied Biosystems (Mulgrave, VIC, Australia) and GeneMarker V1.91 software (SoftGenetics LLC, State College, PA). A panel of 16 markers was tested, and each cell line had a distinct individual set of markers present [25]. Tissue microarrays (TMAs) were constructed from formalin-fixed paraffin-embedded melanocytic tumor tissues retrieved from the Department of Tissue Pathology and Diagnostic Oncology at the Royal Prince Alfred Hospital, Australia (Supplementary Table S1). Studies using human tissues were approved by the Human Research Ethics Committees of the University of Newcastle and Royal Prince Alfred Hospital, Australia.

### Antibodies and reagents

The antibody against Noxa was purchased from Imgenex (San Diego, CA). The antibodies against pCREB1, CREB1, pERK1/2, BRAF, p62, Caspase 3 and cleaved PARP were from Santa Cruz Biotechnology (Santa Cruz, CA); the antibody agaist ERK1/2 was from Cell Signaling Technology (Beverly, MA); The antibodies against LC3-I/II and LC3-II were from Abgent (San Diego, CA). PLX4720 (4 mM stock in DMSO) was from Selleck Inc. (Houston, TX); Actinomycin D (100 μg/ml stock in DMSO), MG132 (10 mM stock in DMSO) and bafilomycin A1 (1 mM stock in DMSO) were from Sigma-Aldrich (Castle Hill, NSW, Australia); U0126 (10 mM stock in DMSO) was from Promega (San Luis Obispo, CA);

### Immunohistochemistry (IHC)

IHC staining and quantitation of immunostained cells were performed as described previously [62]. The Noxa antibody (Imgenex, San Diego, CA) specificity was confirmed by using a blocking peptide that abolished the immunoreactivity in IHC assays.

### CellTiter-Glo assays

The CellTiter-Glo assay was performed with the CellTiter-Glo Luminescent Cell Viability Assay kit according to the manufacturer's instructions (Promega, San Luis Obispo, CA). Luminescence was recorded using Synergy™ 2 multi-detection microplate reader (Biotek, Winooski, VT).

### BrdU proliferation assays

BrdU cell proliferation assays were carried out as described previously [63]. Absorbance was recorded at 450nm using Synergy™ 2 multi-detection microplate reader (Biotek, Winooski, VT).

### Clonogenic assays

Clonogenic assays were performed as described previously [64]. Cells were fixed with methanol and stained with 0.5% crystal violet. The images were captured with Bio-Rad VersaDoc image system (Bio-Rad, Gladesville, NSW, Australia).

### siRNA or shRNA-mediated gene suppression

Small interfering RNAs (siRNAs) constructs used were obtained as the siGENOME SMARTpool reagents (Dharmacon, Lafayette, CO). The siRNA constructs used were: CREB1 siRNAs were purchased from Genepharma (Shanghai, China). ERK1 siGENOME SMARTpool (M-003592-03-0005), ERK2 siGENOME SMARTpool (M-003555-04-0005), and non-targeting siRNA pool (D-001206-13-20) as control. Transfection of siRNAs was carried out as described previously [65]. The shRNA targeting Noxa were purchased from Sigma-Aldrich (Castle Hill, NSW, Australia). Generation of lentiviruses expressing Noxa or control small hairpin RNAs (shRNAs) were as described previously [30].

### Western blot analysis

Western blot analysis was carried out as described previously [35]. Labeled bands were detected by Luminata Crescendo Western HRP substrate (Millipore, Billerica, MA). Images and band intensity were quantitated with ImageReader LAS-4000 (Fujifilm Corporation, Tokyo, Japan).

### Real-time PCR analysis

Real-time PCR was performed using the ABI Fast 7900HT sequence detection system (Applied Biosystems, Foster City, CA) as described previously [66]. Primers for Noxa are 5′-GCTGGAAGTCGAGTGTGCTA-3′ (forward) and 5′-CCTGAGCAGAAGAGTTTGGA-3′ (reverse) [67]. Primers for β-actin are 5′-GGCACCCAGCACAATGAAG-3′ (forward) and 5′-GCCGATCCACACGGAGTACT-3′ (reverse) [34].

### Plasmid vector and transfection

The *Noxa* promoter luciferase reporter constructs were kindly gifts from Dr C Lallemand (Institut Andreé Lwoff, Villejuif, France). The lentiviral vector *pCDH-CMV-MCS-EF1-copGFP* containing Myc-tagged BRAF^V600E^ was used to produce lentiviruses and to transduce HEMn-MP and Mel-RM as described previously [34].

### Dual-luciferase reporter assay

Transient transfection of indicated luciferase reporter plasmids were carried out as described previously [30]. Renilla plasmid was also included in each transfection to normalize the transfection efficiency. Firefly and Renilla luciferase activities were analyzed by Dual-Luciferase Reporter Assay system according to the manufacturer's instructions (Promega, Madison, WI). The relative luciferase activities were calculated by normalizing the firely luciferase activity to Renilla luciferase activity. The represented data were mean±S.E.M. of three independent experiments.

### ChIP assays

IgR3 and Mel-RM cells incubated with or without PLX4720 were crosslinked with 1% formaldehyde for 10 min at room temperature. ChIP assay was performed according to the manufacturer's instructions by using anti-CREB1 and the ChIP assay kit (Millipore, Merck KGaA, Darmstadt, Germany).

### Statistical analysis

Statistical analysis was performed as described previously [64]. In brief, analysis was performed using GraphPad Prism (La Jolla, CA). Student's *t*-test was used to assess differences in the expression of the proteins between different groups. A *p* value less than 0.05 were considered statistically significant.

## SUPPLEMENTARY MATERIAL FIGURES AND TABLE


