# Supplementary material for: Noxa upregulation by oncogenic activation of MEK/ERK through CREB promotes autophagy in human melanoma cells
Source: Oncotarget. 2014 Oct 21;5(22):11237–51. doi: 10.18632/oncotarget.2616 (PMC4294377; doi:10.18632/oncotarget.2616)
Supplement: Supplementary file 1 [file oncotarget-05-11237-s001.pdf]

Noxa upregulation by oncogenic activation of MEK/ERK through CREB promotes autophagy in human melanoma cells

Supplementary Material

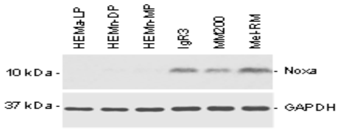

Supplementary Figure S1: Melanocyte lines of different origina express similarly lower levels of Noxa than melanoma cell lines. Whole cell lysates from HEMa-LP, HEMn-DP, HEMn-MP melanocytes and IgR3, MM200, Mel-RM melanoma cells were subjected to western blot analysis. Data shown are representative of three individual experiments.

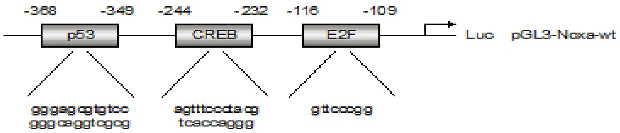

Supplementary Figure S2: A schematic illustration of the bind sites of p53, CREB, and E2F at the *Noxa* promoter.

Supplementary Table S1: Summary of melanocytic tumors and their positivity for Noxa[a]

Melanocytic tumors	Number	Noxa expression levels (IRS)[b]	% Noxa positive cells
Compound Nevus	10	4.2	41[c](21-65)[d]
Dysplastic Nevus	10	4.7	57 (17-90)
Thin Primary (<1mm Breslow depth)	20	5.7	60 (0-90)
Thick Primary (>1mm Breslow depth)	20	8.0	74 (40-97)
Lymph Node Metastases	20	7.9	82 (30-100)
Distant Metastases	20	10.0	72 (0-100)

[a] The cohort of the melanocytic tumors used in this study was the same to that reported before (30).
[b] IRS: Immunoreactive score.
[c] Numbers stand for the mean percentage of positive cells.
[d] Numbers in brackets represent the range of positive cells.
